# Nasal Myiasis in a Female with Christ—Siemens—Touraine Syndrome: A Case Report

**DOI:** 10.31729/jnma.8848

**Published:** 2024-12-31

**Authors:** Leison Maharjan, Anju Shah, Dhirendra Yadav, Namita Shrestha

**Affiliations:** 1 Department of Otolaryngology, Head and Neck Surgery, Patan Academy of Health Sciences, Lagankhel, Lalitpur, Nepal; 2 Department of Otorhinolaryngology, District Hospital, Siddhichardan, Okhaldhunga, Nepal; 3 Patan Academy of Health Sciences, Lagankhel, Lalitpur, Nepal

**Keywords:** *atrophic rhinitis*, *christ-siemens-touraine syndrome*, *hypohidrotic ectodermal dysplasia*, *maggots*, *myiasis*

## Abstract

Ectodermal dysplasia is a rare disease that belongs to a diverse group of inherited monogenic disorders involving defects in one or more ectodermally or mesodermally derived tissues. Hypohidrotic ectodermal dysplasia, also known as Christ-Siemens-Touraine syndrome is a type of ectodermal dysplasia characterized by a triad of anhidrosis, dysodontia, and hypotrichiasis. The most prevalent method of transmission is X-linked recessive, manifesting fully in men and only partially in female carrier heterozygotes. Atrophic rhinitis and nasal myiasis are rare characteristics of this condition. We hereby report a case of a 52-year-old female with atrophic rhinitis and nasal myiasis who was managed conservatively.

## INTRODUCTION

Ectodermal dysplasia (ED) is a group of inherited disorders involving the absence or dysplasia of the ectodermal appendages.^1^ It belongs to a diverse group of monogenic disorders that are characterized by defects in one or more ectodermally or mesodermally derived tissues.^2^ The incidence of ED is estimated at 1:100,000 live births.^3^ Hypohidrotic ectodermal dysplasia (HED), also known as Christ-Siemens-Touraine syndrome, is a type of ED, that is inherited in an X-linked recessive, autosomal dominant, and autosomal recessive manner.^1,2,4^ Typical cases have the characteristic triad of anhidrosis, dysodontia, and hypotrichiasis.^2^ Hereby, we report an unusual case of HED with atrophic rhinitis and nasal myiasis in a female patient.

## CASE REPORT

A 52-year-old female was referred to our hospital with a chief complaint of crawling sensations in the nasal cavity for three days which was associated with episodes of intermittent, blood-mixed, foul-smelling discharge. She gave a classical history of decreased sweating since early childhood, delayed and abnormal teeth eruption, and reduced hair growth. She had also been suffering from anosmia and bilateral nasal obstruction which was associated with frequent mucopurulent foul-smelling nasal discharge since she was a teenager. She has two sisters and a brother. There was no history of similar features in any other family members. Her family history did not reveal any consanguinity.

**Figure 1:**
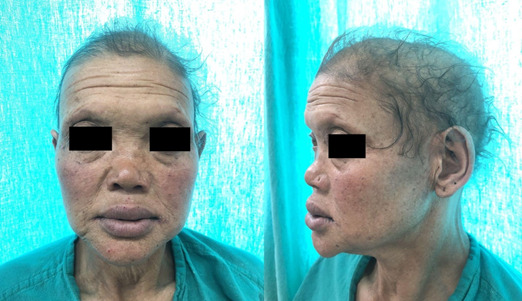
Typical facies of HED. Note the hypohidrotic skin, hypotrichiasis.

**Figure 2:**
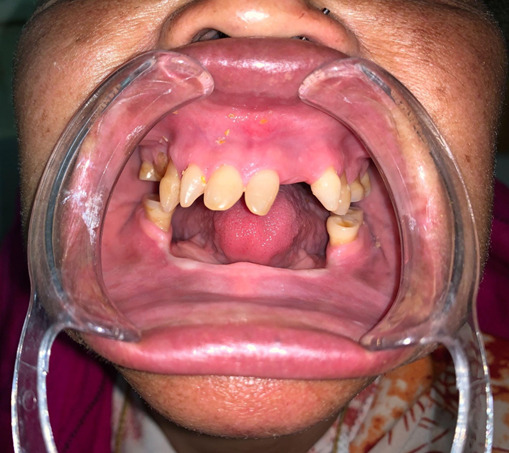
Oligodontia. Note the absence of multiple teeth, conical-shaped maxillary teeth, and attrited molars.

**Figure 3:**
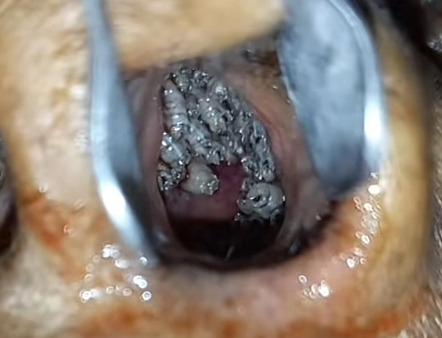
Maggots in the left nasal cavity.

On physical examination, she had characteristic facial features such as a widened and depressed nasal bridge, prominent lips, loss of eyebrow hairs, eyelashes, and markedly reduced scalp and body hairs. The scalp had light-colored, thin, and fragile hair (Figure 1). The skin felt dry and soft with definitive hypohidrosis as proven by a brisk exercise test. She had thick skin on her palms and soles with multiple fissures. Examination of the oral cavity revealed the presence of only 14 teeth (oligodontia)- 10 maxillary teeth and 4 mandibular teeth. Maxillary teeth were malformed-conical shaped and molars were attrited (Figure 2). Examination of the nasal cavity revealed a dull edematous mucosa, and atrophic turbinates with maggots present in the bilateral nasal cavity (Figure 3). On further endoscopic examination, maggots were present in the nasopharynx, inside eustachian tube openings along with the presence of mucopurulent discharge.

**Figure 4:**
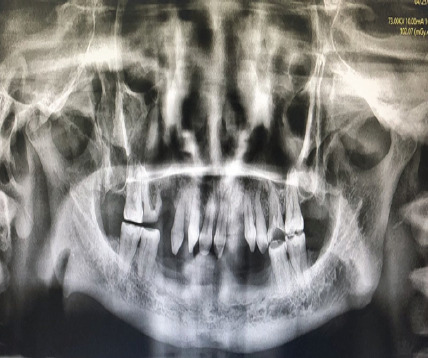
OPG shows oligodontia, conical-shaped maxillary teeth, caries of right upper first molar and attrited molars.

Orthopantomography (OPG) revealed oligodontia, conical-shaped maxillary teeth, caries in the right upper first molar, and attrited molars. (Figure 4). Laboratory investigations revealed a normal hemogram, serum biochemistry, urine analysis, chest radiography, and ECG. A final diagnosis of HED with atrophic rhinitis and nasal myiasis was made based on the clinical and radiological features. She was admitted and started on intravenous Ceftriaxone for her nasal infection. As she also had coexisting oropharyngeal and laryngeal candidiasis, oral fluconazole along with clotrimazole mouth paint was also prescribed. Endoscopic removal of maggots (total of 75+ in count) was performed in multiple settings in local anesthesia. Some maggots were sneezed out by the patient herself. Complete removal of maggots was confirmed with nasal endoscopy. After the treatment, she was discharged on oral antibiotics, saline-alkaline nasal douching, and regular instillation of 25% glucose + glycerine drops. She was also advised to go for prosthodontic treatment. However, patient refused karyotyping.

## DISCUSSION

ED is divided into two categories based on the number and function of sweat glands: hidrotic ectodermal dysplasia (Clouston syndrome) and hypohidrotic/anhidrotic ectodermal dysplasia (Christ-Siemens-Touraine syndrome).^4^ HED was first described by Widderburn in Hindu family in 1838. The term "hereditary ectodermal dysplasia" was first described by Thurnam in 1848 and coined by Weech in 1929.^5^

HED is usually inherited as an X-linked recessive trait (ED1 [MIM 305100]), and rarely as an autosomal dominant (ED3 [MIM 129490]) and an autosomal recessive (MIM 224900) form.^1^

HED exhibits the classic triad of hypohidrosis, hypotrichosis, and hypodontia. The classical syndrome occurs in an X-linked recessive manner which is the most common form of ED and shows complete expression in males, whereas the female carriers show only partial manifestations as heterozygotes.^6^ HED is caused by mutation of a gene that encodes several proteins with roles in the ectodysplasin signal transduction pathway. Mutation in the ectodysplasin-A (EDA) gene is responsible for its X-linked recessive form. Morphogenesis of ectodermally derived structures, especially the epithelial cells of the hair follicles, sweat glands, and developing tooth germ is signaled through this pathway which results in genetic defects leading to aplasia, hypoplasia, or dysplasia of these structures. It also leads to disturbances in the dental organ and enamel matrix formation, ensuing hypodontia and hypoplasia of teeth.^6^ HED may be accompanied by immunodeficiency diseases. Nuclear factor kappa B essential modulator (NEMO) results in X-linked ectodermal dysplasia with immunodeficiency due to its hypomorphic mutations.^1^ In our case, as both of the patient's parents and her siblings (two sisters and a brother) were clinically unaffected, it suggests that she had either autosomal recessive inheritance or a de novo autosomal dominant mutation.

Common features of HED include frontal bossing, thick lips, broad depressed nasal bridge of nose and deformed ears, sparse and fine blond hair with abnormal texture of the scalp, eyebrows, and eyelashes, dry skin, and nail defects.^6^ In addition, patients can experience eczema, otitis media, cerumen impaction, nasal obstruction or crusting, alopecia, heat intolerance, and episodes of hyperpyrexia.^7^ Respiratory system involvement such as atrophic rhinitis, chronic pharyngitis, and recurrent chest infections is attributed to the hypoplasia of the mucous glands.^8^ A study done amongst ED showed a comparatively low prevalence (35%) in females compared to males. In that study of 75 participants, nasal symptoms were particularly problematic, especially nasal obstruction (51%), dryness (49%), and crusting (49%). Furthermore, 40% of patients had previous otolaryngologic surgery, with the most common procedures being bilateral myringotomy and tube placement.^7^ The diagnosis of HED can be confirmed by skin biopsy, which shows absence of sweat glands. Due to which the core temperature increases along with the ambient temperature in these patients, and febrile convulsions might also occur.^8^

Myiasis is defined as an infestation of the ear, nose, and throat with maggots, and fly larvae (genus Chrysomyia). It is commonly seen in tropical regions. The damage from the maggots to the face, nose, and intracranial tissues can even result in meningitis and death.^9^ Atrophic rhinitis with foul-smelling discharge is the main risk factor for nasal myiasis in immune-competent patients.^8,9^ There have been only few reported cases of HED coexisting with atrophic rhinitis and myiasis. Bhat et al. have reported a case of nasal myiasis and aural myiasis with cholesteatoma in the female patient.^9^

An effective method of conservative management involves packing the nose with chloroform or a chloroform and turpentine (1:4) mixture, following which the dead maggots are manually removed. Due to the unavailability of chloroform, turpentine alone can also be used. To avoid secondary infections, antibiotic coverage may be advised. Due to primary defects in the development of structures derived from the embryonic ectoderm, surgical options for atrophic rhinitis are also not entirely satisfactory.^10^ The management of the condition may benefit from the partial closure of both nostrils to enhance the health of the nasal mucosa.^7^ Gene analysis is also advised in such patients whenever available. It also helps in antenatal diagnosis.
